# Recognising and responding to deteriorating patients: what difference do national standards make?

**DOI:** 10.1186/s12913-019-4339-z

**Published:** 2019-09-05

**Authors:** Matthew H. Anstey, Alice Bhasale, Nicola J. Dunbar, Heather Buchan

**Affiliations:** 1 Intensive Care Department, Sir Charles Gairdner Hospital, Level 4 G Block, Hospital Ave, Nedlands, Perth, WA 6009 Australia; 2 School of Public Health, Curtin University, Perth, Australia; 3 Australian Commission on Safety and Quality in Health Care, Sydney, NSW Australia

**Keywords:** Quality improvement, Accreditation of hospitals, Standards, Measurement of quality, Surveys

## Abstract

**Background:**

The Australian Commission on Safety and Quality in Health Care released a set of national standards which became a mandatory part of accreditation in 2013. Standard 9 focuses on the identification and treatment of deteriorating patients. The objective of the study was to identify changes in the characteristics and perceptions of rapid response systems (RRS) since the implementation of Standard 9.

**Methods:**

Cross-sectional study of Australian hospitals. Baseline data was obtained from a pre-implementation survey in 2010 (220 hospitals). A follow-up survey was distributed in 2015 to staff involved in implementing Standard 9 in public and private hospitals (276 responses) across Australia.

**Results:**

Since 2010, the proportion of hospitals with formal RRS had increased from 66 to 85. Only 7% of sites had dedicated funding to operate the RRS. 83% of respondents reported that Standard 9 had improved the recognition of, and response to, deteriorating patients in their health service, with 51% believing it had improved awareness at the executive level and 50% believing it had changed hospital culture.

**Conclusions:**

Implementing a national safety and quality standard for deteriorating patients can change processes to deliver safer care, while raising the profile of safety issues. Despite limited dedicated funding and staffing, respondents reported that Standard 9 had a positive impact on the care for deteriorating patients in their hospitals.

**Electronic supplementary material:**

The online version of this article (10.1186/s12913-019-4339-z) contains supplementary material, which is available to authorized users.

## Background

A number of the deaths that occur in hospitals are known to be preventable [1]. In many cases there is a preceding period when it is clear that the patient’s condition is deteriorating [1]. Early recognition of deterioration followed by appropriate intervention can often prevent death. Many large hospitals around the world have instituted rapid response teams to “rescue” deteriorating patients before more serious complications occur [2–4].

Australia has led the world in setting up hospital based rapid response teams with almost 60% of Australian hospitals with an intensive care unit having a Medical Emergency Team in 2007 (MET) [5, 6]. However, many Australian hospitals lacked comprehensive systems for recognising and responding to clinical deterioration. Where MET were established they were not necessarily fully integrated into hospital systems, and some focused more on responding to crises (just cardiac arrest) than on proactive monitoring for deterioration.

In 2011 the Australian Commission on Safety and Quality in Health Care released a set of ten National Safety and Quality Health Service (NSQHS) Standards [7]. The primary aims of the NSQHS Standards are to protect the public from harm and improve the quality of health service provision. They ensure that relevant systems in place to ensure expected standards of safety and quality are met. The NSQHS Standards were endorsed by all Australian Health Ministers and assessment against the NSQHS Standards has been mandatory for accreditation of all acute health services, both public and private, since 2013 [7].

Within the NSQHS Standards, the Recognising and Responding to Clinical Deterioration in Acute Health Care Standard (the Standard) describes the actions and criteria for a hospital-wide system for recognising and responding to clinical deterioration in hospitalised patients. It is based on a national consensus statement published in 2010 which outlined eight essential elements, including both clinical and organisational processes supporting monitoring of vital signs, detection of deterioration, escalation and rapid response [8]. These elements are reflected in the criteria required to achieve the Standard [6] (Table 1).

**Table 1:** Criteria to achieve Standard 9

Establishing recognition and response systems	
Health service organisations have policies or protocols for the organisation that are implemented in areas such as: measurement and documentation of observations, escalation of care, establishment of a rapid response system and communication about clinical deterioration. Information about these systems is collected and feed back to the clinical workforce to track performance and outcomes over time.	
Recognising clinical deterioration and escalating care	
Mechanisms that record physiological observations and include triggers to escalate care when deterioration occurs are in place. Mechanisms are in place to escalate care and call for emergency assistance.	
Responding to clinical deterioration	
Criteria for triggering a call to the response team are included in the escalation protocol and these calls are regularly reviewed. The clinical workforce is trained in basic life support, and a clinician with advanced life support training is available on-site or nearby at all times.	
Communicating with patients and carers	
Patients, families and carers are informed of recognition and response systems and can respond to the processes of escalating care	

Source: National Safety and Quality Health Service Standards, 2011 [7]

In 2010, as part of the development process for Standard 9, the Commission undertook a survey of rapid response systems involving 220 Australian hospitals [9]. At that time, approximately two thirds of respondents had some form of rapid response system in place. This paper reports on the results of a survey of recognition and response systems conducted in June 2015 which aimed to measure the systems and policies put in place to support Standard 9 as well as the perceived consequences of these on patient outcomes, staffing and workload.

## Methods

We conducted a cross-sectional study of Australian hospitals over a 3-week period in June 2015. The invitation to individual hospitals to participate in the survey was coordinated through two of the Commission’s key committees – the Inter-Jurisdictional Committee (composed of senior safety and quality policymakers from the Australian Government Department of Health and state and territory health departments), and the Private Hospital Sector Committee. The Inter-Jurisdictional Committee distributed the survey to public hospitals through their contact lists. For the private sector, the invitation to participate in the survey was distributed by the Australian Private Hospitals Association and Catholic Health Australia. The intended participants were hospital staff involved with implementing recognition and response systems in their facility. In order to ensure the best possible response rates, the Commission followed up with reminders about distribution but had no direct control over distribution lists or follow up.

To assess change over time, the 2015 survey repeated some questions from the 2010 survey. These questions covered hospital processes for recognising and responding to deterioration (such as the use of track and trigger systems and availability of emergency assistance including medical emergency teams) and organisational systems to support clinical staff (such as the provision of education and clinical audit processes). Questions were added to the 2015 survey to better understand the impact of the Standard and changes required within hospitals to meet the criteria for accreditation.

The survey was conducted using a web-based SurveyMonkey platform (survey available in Additional file 1). The Commission asked those distributing the survey to remind participants 1 week before the survey closed.

## Analysis

Results of the 2010 and 2015 national surveys were compared using descriptive statistics. Significance testing was not performed as the sample of hospitals completing the 2015 survey was different from the 2010 survey. Nonetheless, it was possible to determine that 37 hospitals had completed both surveys. The results of this subgroup were analysed separately and compared to the overall survey results. New South Wales hospitals participated in the 2015 survey, but not in the 2010 survey (due to parallel evaluation processes being undertaken as part of the New South Wales Between the Flags Program).

## Results

### Response rate and characteristics

After excluding duplicate responses and responses from day procedure services (which were not the target audience for the survey) there were 276 responses to the 2015 survey. As the distribution occurred at the jurisdictional/private hospital association level to protect anonymity, details of the number of hospitals invited to participate were not available and a true response rate could not be calculated. At the time of the survey, there were 724 public, and 286 private hospitals (excluding day hospital facilities) in Australia [10]. Not all 276 participants answered all survey questions.

Of the participating hospitals, 147 (71%) were public. The size of participating hospitals ranged from under 10 to over 500 beds. Individuals who completed the survey varied considerably, and included safety and quality professionals, clinical managers, clinical educators, medical and nursing professionals and executives.

### Changes in systems from 2010

**Fig. 1:**
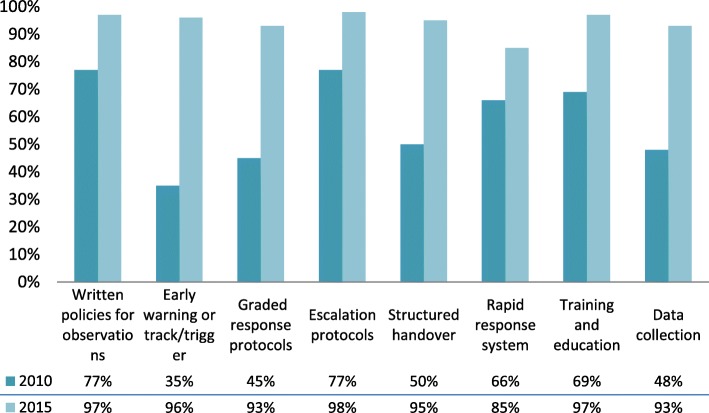
Features of recognition and response systems, 2010 and 2015. Description: A comparison of the features of recognition and response systems between the two time periods. Note: percentages are based on the number of respondents to each question, excluding missing data

There were changes in the characteristics and composition of recognition and response systems between 2010 and 2015 that were in line with the requirements of the Standard. These changes are outlined in Fig. 1, Tables 2 and 3.

**Table 2:** Features of recognition and response systems, 2010 and 2015

	2010 *n* = 220 (%)	2015 *n* = 276 (%)
Written policies for observations	168 (77%)	270 (97%)
Minimum frequency and observations required	106 (63%)	243 (96%)
Policy applies to all patients	139 (82%)	248 (97%)
Escalation protocols	170 (77%)	256 (98%)
Includes a graded response	76 (45%)	230 (93%)
Early warning systems or track/trigger	77 (35%)	244 (96%)
Track and trigger actions incorporated into chart	30 (39%)	195 (81%)
Single or multi-parameter systems	45 (58%)	169 (71%)
Combined system (calling criteria and score)	20 (26%)	38 (16%)
Required calculation of a score (such as MEWS)	8 (10%)	11 (5%)
Origin of chart used^a^		
State chart	–	125 (50%)
Australian Commission on Safety and Quality in Health Care	–	69 (28%)
Local chart	–	33 (13%)
Combination	–	23 (10%)
Use structured protocol for handover communication	110 (50%)	237 (95%)

Percentages are percent of those responding for that category

^a^Multiple responses possible in each category

**Table 3:** Location and composition of rapid response systems

	2010 n (%)	2015 n (%)
Any formal rapid response system	145/220 (66%)	207/244 (85%)
In hours system	(*n* = 145)	(*n* = 204)
Based in ICU	29 (20%)	46 (23%)
Based outside ICU	56 (39%)	82 (40%)
Emergency department	19	33
Medical units	18	35
Other^b^	19	14
Combination of departments	31 (21%)	51 (25%)
External to hospital	29 (20%)	25 (12%)
Ambulance	9	14
Visiting medical officers	4	6
GPs	12	3
Other^b^	4	2
Out of hours system	(*n* = 145)	(*n* = 196)
Based in ICU	29 (20%)	47 (24%)
Based outside ICU	56 (39%)	121 (62%)
External to hospital	34 (23%)	28 (14%)
Combination of departments	26 (18%)	~
Leaders of rapid response system^c^		
When based in ICU		
Doctors	35 (68%)	33 (77%)
Nurses	16 (13%)	11 (22%)
When based outside ICU		
Doctors	27 (61%)	40 (56%)
Nurse	14 (28%)	32 (44%)
People who can activate rapid response system		
Nurses	147 (100%)	186 (100%)
Doctors	130 (89%)	161 (87%)
Other hospital staff	101 (69%)	158 (85%)
Family, patients and carers	26 (18%)	105 (56%)

^b^Other or missing

^c^In hours leaders only. May not sum to 100% due to “other” responses

^~^ 2015 survey required participants to select one option only, so combination not possible here

#### Organisational systems to support the recognition of and response to deterioration

In 2015, 85% of respondents had a formal rapid response system (as distinct from a code blue or “cardiac arrest” only team), compared with 66% in 2010. Those who did not have a separate system were predominantly from small hospitals (9 hospitals with less than 10 beds, 11 hospitals with less than 50 beds).

Different variations of rapid response teams existed in both 2010 and 2015, from all the clinical staff on each ward, to specialised teams. However in 2015, there appeared to be fewer systems based solely in the intensive care unit (ICU), although the number of hospitals with an ICU was similar in the two surveys.

Changes were also seen in the proportion of hospitals that allowed rapid response systems to be activated by non-clinicians with a particularly large difference for patient and family activation (56% 2015 vs 18% in 2010).

Similar improvements were seen when results from the sub-sample of 37 hospitals participating in in both surveys were compared with the overall 2015 results.

#### Funding and staff allocation

In 2015, 81% of respondents had specific staff responsible for implementing and monitoring the operation of recognition and response systems. Most services implemented these systems with less than 0.5 full time equivalent (FTE), and only 7% received specific funding for operating the rapid response system (Table 4).

**Table 4:** Resourcing for recognition and response systems

	2010 n (%)	2015 n (%)
Dedicated staff for recognition and response systems monitoring/implementation	152/220 (69%)	186/244 (76%)
Staffing allocation for monitoring/implementation		
< 0.5 FTE	102/152 (67%)	115/186 (62%)
> 1 FTE	18/152 (12%)	13/186 (7%)
Specific funding for operation of rapid response system	12/152 (6%)	15/186 (8%)

#### Training and education

Training and education in managing clinical deterioration had increased by 2015. In 2015, 97% of respondents reported that their hospital provided regular training and education to support staff in the recognition of and response to clinical deterioration, compared with 69% in 2010. The types of education and training provided in 2015 are outlined in Table 5.

**Table 5:** Type of education and training provided in 2015

Type of training provided by the health service	For doctors *n* = 188 n (%)	For nurses *n* = 204 n (%)	Other hospital staff *n* = 178 n (%)
Orientation training about existence of rapid response system and how to call	172 (92%)	200 (98%)	143 (80%)
Basic life support	149 (82%)	204 (99%)	163 (89%)
Advanced life support	139 (75%)	176 (88%)	5 (3%)
Measurement and interpretation of observations	107 (61%)	197 (97%)	28 (18%)
Management of deteriorating patients	143 (79%)	201 (97%)	65 (40%)
Communication skills	105 (61%)	176 (88%)	97 (59%)
Team work	100 (60%)	159 (82%)	99 (60%)

#### Governance, monitoring and feedback

In 2015, 86% of respondents had a governance committee for recognition and response systems compared with 72% in 2010, while 84% reported regularly to the executive, compared with 65% in 2010. One of the requirements in the Standard includes collecting information, providing feedback to the clinical workforce, and tracking outcomes and changes in performance over time. Almost all respondents to this question in 2015 (93%) reported that their hospital collected specific data about the effectiveness of their recognition and response systems. This is almost double the proportion in the 2010 survey (48%). Data routinely collected included audits of completion of observation charts (81%), numbers of rapid response calls (71%), number of cardiac arrests (69%), audits of failure to escalate (45%), number of calls for ward review (45%), number of patients with a not for resuscitation order (33%), and number of unplanned ICU admissions (32%). Reports were created to provide feedback to wards (67%), put on the hospital intranet (20%) and some were made publicly available (6%).

#### Perceptions of the standard

Respondents to the 2015 survey were asked “Do you believe that the Standard has improved the recognition of, and response to deteriorating patients in your health service?” A large majority (83%) of respondents answered positively “yes” (13% unsure, 5% no). Of those who responded yes, the reasons provided were included improved monitoring of vital signs (69%), more frequent escalation for patients with deteriorating vital signs (66%), better management of deteriorating patients on the ward (64%), improved awareness at executive level (51%), change in hospital culture (50%), deteriorating patients more likely to be transferred to another facility (38%), improved staffing (3%), empowerment of nursing staff (3%).

For the 5% of respondents who said that the Standard had not improved recognition and response to deteriorating patients, this was either because they already had a system in place, or they felt that the Standard increased paperwork and reduced the place for clinical judgment. Some respondents pointed to the difficulties of implementing the Standard for small organisations and mentioned that managing end-of-life care is an ongoing issue.

#### Public hospitals compared with private hospitals

For the 2015 survey there were no differences between respondents at public or private hospitals for most responses. Significantly, there were no differences between public and private hospitals in their perceptions of whether the Standard had improved the recognition and response to deteriorating patients. However, private hospital respondents were less likely to report training in advanced life support for doctors (55% for private hospitals vs 81% for public hospitals), basic life support for doctors (68% vs 87%) or advanced life support training for nurses (74% vs 92%).

## Discussion

The majority of respondents believed that the Standard had improved the recognition of, and response to deteriorating patients in their health service. The results suggest that the Standard has reinforced the importance of properly recognising and responding to clinical deterioration in a systematic way, and supported changes to the culture of organisations. There appears to be a greater focus on measuring performance about how deteriorating patients are recognised and responded to, learning for improvement, and on engaging clinicians across a hospital to recognise and respond to deterioration.

These improvements have occurred even though 66% of hospitals already had some form of rapid response system prior to the implementation of the NSQHS Standards. The responses suggested that this was at least partly due to prioritisation of this issue by hospital executives, and widespread educational efforts that helped to alter the hospital culture to focus on the importance of recognising and responding to deteroriating patients. Furthermore, the additional staffing and funding provided for the implementation and monitoring of the Standard was minimal. This suggests that sites were making use of already employed staff, and where necessary, staff may have been re-assigned to work on the Standard, as the introduction of the NSQHS Standards increased the priority recognising and responding to deterioration for health services. Our survey does not provide any information about whether there was an opportunity cost in re-directing staff from other tasks.

Data on the outcomes of accreditation reported to the Commission show that the proportion of hospitals meeting both core and developmental requirements of the Standard at first assessment increased between 2013 and 2015. For example, in 2013 54% of hospitals met developmental actions while 78% did in 2015; and 97% met core actions increasing to 100% at first assessment in 2015. While participant-reported outcomes are not the same as objective improvements, recent evidence has suggested an association between implementation of the Standard and health outcomes such as decreased rates of in-hospital cardiac arrests, reduced cardiac arrest-related ICU admissions and subsequent in-hospital mortality [11, 12].

Aspirational statements about patient safety will not, on their own, ensure that safe care is consistently and reliably delivered. Unless organisations have systems and processes in place that are supported by governance bodies, quality and safety efforts can be sporadic, confined to specific services or dependent on the enthusiasm and determination of individuals. Defined standards that apply across all health care organisations which identify evidence-based systems and processes may promote large scale change and encourage local, related improvement efforts. However, previous reviews have suggested that the evidence underpinning accreditation standards is lacking, but it may be that the way that accreditation programs are developed affects their success [13–15]. Respondents to this survey were positive about the effects of a system-wide mandated standard. The clinical evidence underpinning rapid response teams have been established, but the Standard allowed their spread to all acute hospitals [16, 17]. Overall, 83% of respondents felt that the Standard has had a positive impact on the care of deteriorating patients in hospitals, despite limited dedicated funding to achieve this. Because the NSQHS Standards are mandatory for all types of hospitals, hospitals without ICUs, and sometimes without full-time medical coverage now also need to have systems in place. More hospitals now have recognition systems in place such as early warning track and trigger tools and graded response protocols.

## Limitations

This was not a research study and the survey methodology has several limitations. First, the results presented are the perceptions of staff, rather than measures of patient outcomes. The role of the respondent may also influence how positive or negative they were in their perception of the Standard, as well as their eagerness to provide a positive response to a national organisation (the Commission) [18, 19]. However other research has suggested that the Standard has improved patient outcomes, and this survey was designed to understand the extra workload placed on hospitals to achieve this [11, 12].

Second, New South Wales did not participate in the 2010 survey. However, when the analyses with the whole 2015 sample were compared to the analyses without New South Wales, there were no differences of note (Table 6). Third, since not all the information is available about the hospitals participating in each survey, it is not possible to know whether the two samples are equivalent. However, when we compared the hospitals that we knew participated in both surveys, the results were similar to those overall. While the Standard and the consensus statement were the only national initiatives between 2010 and 2015, some state and territory health departments and individual hospital initiatives may have had their own initiatives in the area of managing clinical deterioration (for example Between the Flags in New South Wales). Importantly, these initiatives are aligned in purpose and goal, as the NSQHS Standards were developed in collaboration with state and territory representatives taking into account their safety and quality priorities. Finally, the differing roles of respondents may have affected the results.

**Table 6:** Sample of 37 matched hospitals (68% public) responding in 2010 and 2015

	2010	2015
Written observation policy	62%	100%
Policy for actions deteriorating patients	70%	100%
Early warning or track and trigger chart	43%	100%
Structured handover tool	57%	97%
Rapid response team (not code blue)	78%	84%
Staff for monitoring RRS	62%	95%
Committee with oversight RRS	62%	95%
Regular training & education	68%	97%
Collect data on effectiveness RRS	59%	97%
Executive receive regular reports	49%	92%

## Conclusions

Evidence supporting the efficacy of rapid response teams on their own has been mixed [16, 20–23]. However, the Standard covers a far wider range of organisational systems for recognising and responding clinical deterioration. This survey provides supportive evidence that institutional changes in processes have occurred following implementation of the Standard. While there are challenges to achieving standardisation across different sectors within the Australian health system, specifying safety and quality standards whose implementation is linked with maintaining hospital accreditation, appears to provide a valuable lever for change.

## Additional file

Additional file 1: Survey of Recognition and Response Systems: This is a copy of the electronic survey given to respondents. (PDF 753 kb)

## Data Availability

The data that support the findings of this study are available from the corresponding author MA but restrictions apply to the availability of these data, and so are not publicly available. Data are however available from the authors upon reasonable request and with permission of the Australian Commission on Safety and Quality in Health Care.

## Abbreviations

FTE: Full time equivalent
ICU: Intensive care unit
MET: Medical emergency team
NSQHS: National Safety and Quality Health Service
RRS: Rapid response systems
