# Supplementary material for: Recognising and responding to deteriorating patients: what difference do national standards make?
Source: BMC Health Serv Res. 2019 Sep 5;19:639. doi: 10.1186/s12913-019-4339-z (PMC6728974; doi:10.1186/s12913-019-4339-z)
Supplement: Supplementary file 1 — Survey of Recognition and Response Systems: This is a copy of the electronic survey given to respondents. (PDF 753 kb) [file 12913_2019_4339_MOESM1_ESM.pdf]

Survey of Recognition and Response Systems

Background

Recognition and response systems are systems put in place to support staff to promptly and reliably recognise patients who are clinically deteriorating, and to respond appropriately to stabilise the patient.

PURPOSE OF THIS SURVEY
This survey is being conducted by the Australian Commission on Safety and Quality in Health Care as part of its evaluation of the impact of NSQHS Standard 9: Recognising and Responding to Clinical Deterioration on processes and outcomes of care. Its purpose is to obtain information about recognition and response systems in Australian public and private hospitals, and compare the results to those from a similar survey conducted in 2010. The survey collects information about systems, policies and support for recognising and responding effectively to deteriorating patients.

USE OF DATA COLLECTED IN THE SURVEY
Data collected as part of this survey will be held by the Commission. Grouped, de-identified data will be provided to State and Territory Government health departments, and will be presented in a report to the Australian Government Department of Health. No individual hospitals will be identified.

CONFIDENTIALITY
While we ask for the name of your health service to check for multiple responses from the same hospital and to follow-up non-respondents, your answers will be de-identified and will not be attributed to you or your service. The name of your health service will be kept confidential. Any information which links the name of your health service with the data your health service provides as part of this survey will be treated as confidential information. We will not disclose such confidential information without the consent of your health service.

HOW TO FILL IN THIS SURVEY
One survey should be completed for each hospital.This may include one physical site, or a multi-campus hospital where the systems are the same across campuses. Where recognition and response systems are different across campuses, separate surveys should be completed for each campus.

The survey should be completed by a person or team of people who are familiar with the recognition and response systems in place within the hospital. People who may need to be involved in this process could include hospital managers, directors of clinical governance, directors of nursing, directors of medical services, directors of intensive care and CPR committee members.

Your responses will be saved as you complete each page. You can return to the survey later to complete your answers by opening the survey link in the email. Once you submit the survey, no further changes are possible

Thank you for completing the survey.

NSQHS STANDARDS
A better way to care

Survey of Recognition and Response Systems

Systems for recognising clinical deterioration

* 1. Does your hospital have written policies, protocols or guidelines regarding the measurement of physiological observations such as temperature, respiratory rate and blood pressure?

- Yes
- No
- Don't know

NSQHS STANDARDS
A better way to care

Survey of Recognition and Response Systems

2. Does the policy, protocol or guideline apply to patients in general ward areas?

- Yes
- No

3. Does the policy, protocol or guideline specify that observations should be taken on all patients?

- Yes
- No

4. Does the policy, protocol or guideline specify the minimum frequency and type of observations required for patients?

- Yes
- No

NSQHS STANDARDS
A better way to care

Survey of Recognition and Response Systems

* 5. Does your hospital have a formal, written policy or protocol that describes actions that should be taken when abnormal observations or other clinical deterioration is observed?

- Yes
- No
- Don't know

NSQHS STANDARDS
A better way to care

Survey of Recognition and Response Systems

6. Does this protocol include a graded response linked to the level of identified deterioration or length of time of deterioration?

- Yes - different actions are required for different levels or length of observed deterioration
- No - there is only one response for all instances of observed deterioration

7. “Early warning” or “track and trigger” systems are formal systems that rely on routine periodic measurement of observations (tracking), with a predetermined action (trigger) when a certain threshold is reached. These systems can be built into observation charts.
Does your hospital use a formal, documented early warning or track and trigger system?

- Yes
- No
- Don't know

Survey of Recognition and Response Systems

8. Which of the following best describes the early warning or track and trigger system (pick one)?

- If one or more of a number of specific criteria are met, a call for emergency assistance is made (such as the Medical Emergency Team (MET) criteria)
- A score needs to be calculated from a number of specific criteria to determine whether emergency assistance is called (such as Modified Early Warning System (MEWS))
- A combined system (such as both MET and MEWS)
- Other (please specify)

9. Are the triggers for abnormal observations and the response required built into the design of your general observation chart?

- Triggers or cut off scores to indicate abnormality are included on the observation chart
- Actions required in response to abnormality are included on the observation chart
- Both triggers and actions are included on observation chart
- Neither triggers nor actions are included on observation chart

NSQHS STANDARDS
A better way to care

Survey of Recognition and Response Systems

10. What is the origin of your general observation chart?

- Use an observation chart developed by the state or territory health department
- Use one of the observation and response charts developed by the Australian Commission on Safety and Quality in Health Care
- Use an observation chart developed locally
- Other (please specify)

NSQHS STANDARDS
A better way to care

Survey of Recognition and Response Systems

11. Does your hospital use a structured protocol or tool for handover communication? (such as Situation, Background, Assessment, Recommendation - SBAR)

- ◯ Yes
- ◯ No
- ◯ Don't know

NSQHS STANDARDS
A better way to care

Survey of Recognition and Response Systems

12. What is the name of this protocol? (tick as many as needed)

- ☐ SBAR
- ☐ ISOBAR
- ☐ ISBAR
- ☐ SHARED
- ☐ Other (please specify)

NSQHS STANDARDS
A better way to care

Survey of Recognition and Response Systems

13. Are there any comments that you wish to make about the systems you have in place for recognising clinical deterioration?

Survey of Recognition and Response Systems

Systems for responding to clinical deterioration

Rapid response systems are systems for providing emergency assistance to patients whose condition is deteriorating. The system will include the clinical team or individual providing the emergency assistance, and may include on-site and off-site personnel.

* 14. Apart from a cardiac arrest team, does your hospital have a formal rapid response system in place for providing emergency assistance to patients whose condition is deteriorating?

- ○ Yes (this includes hospitals where the cardiac arrest team also provides care to patients who are deteriorating, but have not had a cardiac arrest)
- ○ No
- ○ Don't know

Survey of Recognition and Response Systems

15. During the 'in hours' period, what type of system in place?

- ◯ Rapid response system based in intensive care
- ◯ Rapid response system based outside of intensive care (such as emergency department or acute medical unit)
- ◯ Rapid response system that is external to the hospital
- ◯ Other

For other arrangement (please specify)

NSQHS STANDARDS
A better way to care

Survey of Recognition and Response Systems

16. For rapid response systems based in intensive care

- ◯ This service is led by doctors
- ◯ This service is led by nurses
- ◯ Other

For other arrangement, please specify

NSQHS STANDARDS
A better way to care

Survey of Recognition and Response Systems

17. For rapid response systems based outside intensive care, please complete the following questions regarding the location and leadership of this service:

	Location	Leader
Where is the service located and who leads it?		

Other (please specify)

NSQHS STANDARDS

A better way to care

Survey of Recognition and Response Systems

18. For rapid response systems that are external to the hospital, who provides this service?

- Visiting medical officer
- Local general practitioner
- Local ambulance
- Other

If other - please specify

NSQHS STANDARDS

A better way to care

Survey of Recognition and Response Systems

19. After hours, what type of system in place?

- ○ Rapid response system based in intensive care
- ○ Rapid response system based outside of intensive care (such as emergency department or acute medical unit)
- ○ Rapid response system that is external to the hospital
- ○ No formal system in place
- ○ Other

For other arrangement (please specify)

Survey of Recognition and Response Systems

20. For AFTER HOURS rapid response systems based in intensive care:

- ○ This service is led by doctors
- ○ This service is led by nurses
- ○ Other

If other , please specify

Survey of Recognition and Response Systems

21. For AFTER HOURS rapid response systems based outside intensive care please complete the following questions regarding the location and leadership of this service:

	Location	Leader
Where is the service located and who leads it ?		

Other (please specify)

NSQHS STANDARDS
A better way to care

Survey of Recognition and Response Systems

22. For AFTER HOURS rapid response systems that are external to the hospital, who provides this service?

- Visiting medical officer
- Local general practitioner
- Local ambulance
- Other

For other arrangements, please specify

23. Does anybody else respond to the emergency call (e.g. within the hospital)

- No
- Yes (please specify)

NSQHS STANDARDS
A better way to care

Survey of Recognition and Response Systems

24. Who are the other members of the team that responds to the rapid response or emergency call? (this refers to people assigned to respond to the emergency call, apart from the leader you already identified)

25. Who can call your rapid response system for assistance for patients whose condition is deteriorating? (can tick more than one)

- [ ] Nurses on the ward
- [ ] Doctors on the ward
- [ ] Other hospital staff (such as allied health professionals, ancillary staff)
- [ ] The patient, the patient’s family or carer
- [ ] Other (please describe)

26. Are there any comments you would like to make about the systems your hospital has in place to respond to patients whose condition is deteriorating?

NSQHS STANDARDS
A better way to care

Survey of Recognition and Response Systems

Organisational systems to support the recognition of and response to deterioration

* 27. Within your hospital, are there any staff who have primary responsibility for developing, implementing, sustaining and monitoring your recognition and response systems? (These staff may also have other responsibilities)

- ○ Yes
- ○ No
- ○ Don’t know
- ○ Not applicable – do not have recognition and response systems in place

NSQHS STANDARDS
A better way to care

Survey of Recognition and Response Systems

28. If yes, how many full-time equivalents (FTE) are allocated to this role?

- Less than 0.5 FTE
- 0.5 FTE
- 0.6 – 1 FTE
- More than 1 FTE

NSQHS STANDARDS

A better way to care

Survey of Recognition and Response Systems

29. How many full-time equivalents (FTE) are allocated to providing the rapid response (emergency response) service?

- Less than 0.5 FTE
- 0.5 FTE
- 0.6 – 1 FTE
- More than 1 FTE

* 30. Within your hospital, is there specific funding allocated to the operation of your rapid response system?

- Not applicable – do not have rapid response system in place
- Don't know
- No, emergency assistance provided by the rapid response system is delivered as part of existing services
- Funding for purposes other than staffing
- Funding for staff

If funding is provided for staff, please specify number of FTE funded

NSQHS STANDARDS

A better way to care

Survey of Recognition and Response Systems

31. Is there a committee that oversees the operation of your recognition and response systems? (This committee may also have other responsibilities, or may be separate from existing committees such as cardiac arrest or resuscitation committees.)

- Yes
- No
- Don’t know
- Not applicable – do not have recognition and response systems in place

NSQHS STANDARDS
A better way to care

Survey of Recognition and Response Systems

* 32. Does your hospital provide regular training and education to support staff in the recognition of and response to clinical deterioration?

- Yes
- No
- Don't know

NSQHS STANDARDS
A better way to care

Survey of Recognition and Response Systems

33. What type of education and training is provided?

	Doctors	Nurses	Other staff
Orientation training about existence of rapid response system and how to call			
Basic life support			
Advanced life support			
Measurement and interpretation of observations			
Management of deteriorating patients			
Communication skills			
Team work			

For other staff, please specify who the other staff are

Survey of Recognition and Response Systems

* 34. Do you believe that Standard 9 has improved the recognition of, and response to deteriorating patients in your health service?

- Yes
- No
- Unsure

Survey of Recognition and Response Systems

35. In what ways (tick as many as apply)

- [ ] Change in hospital culture
- [ ] Greater awareness at executive level
- [ ] Improved monitoring of vital signs
- [ ] More frequent escalation for patients with deteriorating vital signs
- [ ] Deteriorating patients are more likely to be transferred to another facility (e.g. hospital with an ICU)
- [ ] Better management of deteriorating patients on the ward
- [ ] Improved staffing
- [ ] Other
- [ ] Other (please specify)

36. How do you know that this improvement has occurred ? Please provide examples or evidence that tells you a change has taken place.

NSQHS STANDARDS
A better way to care

Survey of Recognition and Response Systems

37. Why not?

NSQHS STANDARDS
A better way to care

Survey of Recognition and Response Systems

38. Does your facility collect specific data regarding the effectiveness of your recognition and response systems?

- Yes
- No
- Don’t know

NSQHS STANDARDS

A better way to care

Survey of Recognition and Response Systems

39. Which measures do you systematically document? (can tick more than one)

- Number of calls to the rapid response team
- Number of cardiac arrests
- Number of requests for urgent review
- Number of unplanned admissions to intensive care
- Number of cardiac arrest calls for a patient with a "not for resuscitation order"
- Number of rapid response calls that lead to a transfer to ICU or another hospital
- Number of "failure to escalate" episodes
- Audit the completion of observation charts
- Other (please specify)

40. Does your executive receive regular reports on the operation and outcomes of your recognition and response systems?

- Yes
- No
- Don’t know
- Not applicable – do not have recognition and response systems in place

NSQHS STANDARDS

A better way to care

Survey of Recognition and Response Systems

41. How frequently does the executive receive regular reports on the operation (number of calls, location etc) and outcomes of your recognition and response systems?

- ○ Daily
- ○ Weekly
- ○ Monthly
- ○ Quarterly
- ○ 6-monthly
- ○ Yearly

Other (please specify)

42. What do they do with these reports? (can tick more than one)

- ☐ Make available on hospital intranet
- ☐ Feedback to local ward teams
- ☐ Feedback to teams providing emergency assistance
- ☐ Feedback to individual departments
- ☐ Make available to the public on the hospital website
- ☐ Other (please specify)

* 43. Do you think your organisation would be willing to contribute a limited data set to a national "deteriorating patient" registry?

- ○ Yes
- ○ No
- ○ Don't know

NSQHS STANDARDS

A better way to care

Survey of Recognition and Response Systems

44. Are there any comments you would like to make about the organisational systems your hospital has in place to support the recognition of and response to patients whose condition is deteriorating?

45. Are there any comments you would like to make about Standard 9 itself?

Survey of Recognition and Response Systems

Demographic information

46. What is your hospital name?

Note: The name of your hospital will be used only to count responses and to check for multiple responses from the same hospital. Data will be analysed and reported in a de-identified manner.

* 47. What is your type of hospital?

- Public
- Private
- Public hospital under private management contract

* 48. What is your organisation's Remoteness Area (RA) classification?

- Major city (RA1)
- Inner regional (RA2)
- Outer regional (RA3)
- Remote (RA4)
- Very remote (RA5)
- Unsure (please record your hospital postcode)

Postcode

* 49. What is the average number of available beds?

Number of beds

* 50. Does your hospital have: (tick as many as applicable)

- A general intensive care unit (integrated medical/surgical including ICU managed high dependency unit)
- Integrated intensive care / coronary care / high dependency unit
- Paediatric ICU
- High dependency / step down / special care unit
- No intensive care or high dependency units

Other ICU (please specify type)

51. If there is an intensive care unit, what is the functional ICU level: (as per CICM guidelines)

- ☐ Level 3 (tertiary referral unit)
- ☐ Level 2 (able provide general ICU including renal replacement therapy)
- ☐ Level 1 (able provide short-term cardio-respiratory support)

52. What is the average number of available beds?

General ICU: __________

Other ICU: __________

HDU managed by ICU: __________

Coronary care managed by ICU: __________

53. Does your hospital have on-site medical coverage 24/7?

- ○ Yes
- ○ No

54. If no, please describe the internal and external arrangements in place for providing care to your patients: (tick as many as applicable)

- ☐ On-call visiting medical officers
- ☐ On-call junior medical officers
- ☐ On-call general practitioners
- ☐ Local ambulance service
- ☐ Royal Flying Doctor Service
- ☐ Telephone or other tele-health service to regional or other facility providing higher level of care
- ☐ Telephone or other tele-health service to other support service or network

Other (please specify)

____

NSQHS STANDARDS

A better way to care

Survey of Recognition and Response Systems

Baseline data

The following questions are not for the purposes of evaluation, but to gauge the amount of work occurring on deteriorating patients in different hospitals.

55. How many calls for emergency assistance (such as MET calls) occurred in 2013 & 2014?

2013 __________

2014 __________

56. How many admissions (separations) did your hospital have in

2013 __________

2014 __________

NSQHS STANDARDS
A better way to care

Survey of Recognition and Response Systems

END

Thank you for completing the survey.
If you have any questions or feedback, please contact:

Alice Bhasale
Senior Project Officer
Australian Commission on Safety and Quality in Health Care
email: alice.bhasale@safetyandquality.gov.au
phone: 02 9126 3515
